# Editorial: Digital hearing healthcare

**DOI:** 10.3389/fdgth.2022.959761

**Published:** 2022-07-13

**Authors:** Qinglin Meng, Jing Chen, Changxin Zhang, Jan-Willem A. Wasmann, Dennis L. Barbour, Fan-Gang Zeng

**Affiliations:** ^1^Acoustics Laboratory, School of Physics and Optoelectronics, South China University of Technology, Guangzhou, China; ^2^n^3^ Hearing Laboratory, Guangzhou, China; ^3^Key Laboratory of Machine Perception (Ministry of Education), School of Artificial Intelligence, Speech and Hearing Research Center, Peking University, Beijing, China; ^4^National Biomedical Imaging Center, College of Future Technology, Peking University, Beijing, China; ^5^Faculty of Education, East China Normal University, Shanghai, China; ^6^Department of Otorhinolaryngology, Donders Institute for Brain, Cognition and Behaviour, Radboud University Medical Center Nijmegen, Nijmegen, Netherlands; ^7^Laboratory of Sensory Neuroscience and Neuroengineering, Department of Biomedical Engineering, Washington University in St. Louis, St. Louis, MO, United States; ^8^Department of Otolaryngology - Head and Neck Surgery, Center for Hearing Research, University of California, Irvine, Irvine, CA, United States

**Keywords:** digital hearing healthcare, computational audiology, tele-audiology, audiometry, hearing aid, hearing loss, cochlear implant, tinnitus

The Digital Hearing Healthcare or DHH Research Topic consists of 30 articles using modern digital methods to address a wide range of interesting and important hearing healthcare related issues. The interdisciplinary nature of the DHH Research Topic spans five Frontiers journals and six Frontiers sections. As the host journal, Frontiers in Digital Health published 16 articles, while Frontiers in Neuroscience, Medicine, Psychology, Public Health, and Neurology published 7, 3, 2, 1, and 1 articles, respectively. The Research Topic was initiated in March 2020, opened for submission from August 2020 to Octobor, 2021, with a total of 38 submissions being received.

The World Health Organization (WHO) estimates that “more than 1.5 billion people experience some decline in their hearing capacity during their life course, of whom at least 430 million will require care,” but only a fraction of them are receiving the care, with unaddressed hearing loss resulting in an annual global cost of US$980 billion (1). We believe that digital health methods will play a significant role not only in the prevention, diagnosis, treatment and rehabilitation of hearing loss, but also in increasing the universal coverage of and decreasing the cost and burden of hearing healthcare. Here the term “Digital” is used to signify a much broader context than the traditional “digital signal processing” concept. Digital Hearing Healthcare uses a wide range of digital technologies to address hearing healthcare problems in ways complementary to, or different from, the conventional clinical processes in hospitals or clinics. This Editorial summarizes the key findings and contributions of the DHH Research Topic.

To show the full scope of the Research Topic, Figure 1 displays the 30 articles (middle column) that are conceptually categorized according to the target hearing healthcare issues (left column) and digital techniques (right column). The hearing health issues include basic audiometry tests, hearing devices, advanced hearing ability, and tinnitus therapeutics. The digital techniques include machine learning and big data, smartphone and wearables, tele-audiology, automatic speech recognition, and signal processing.

**Figure 1:**
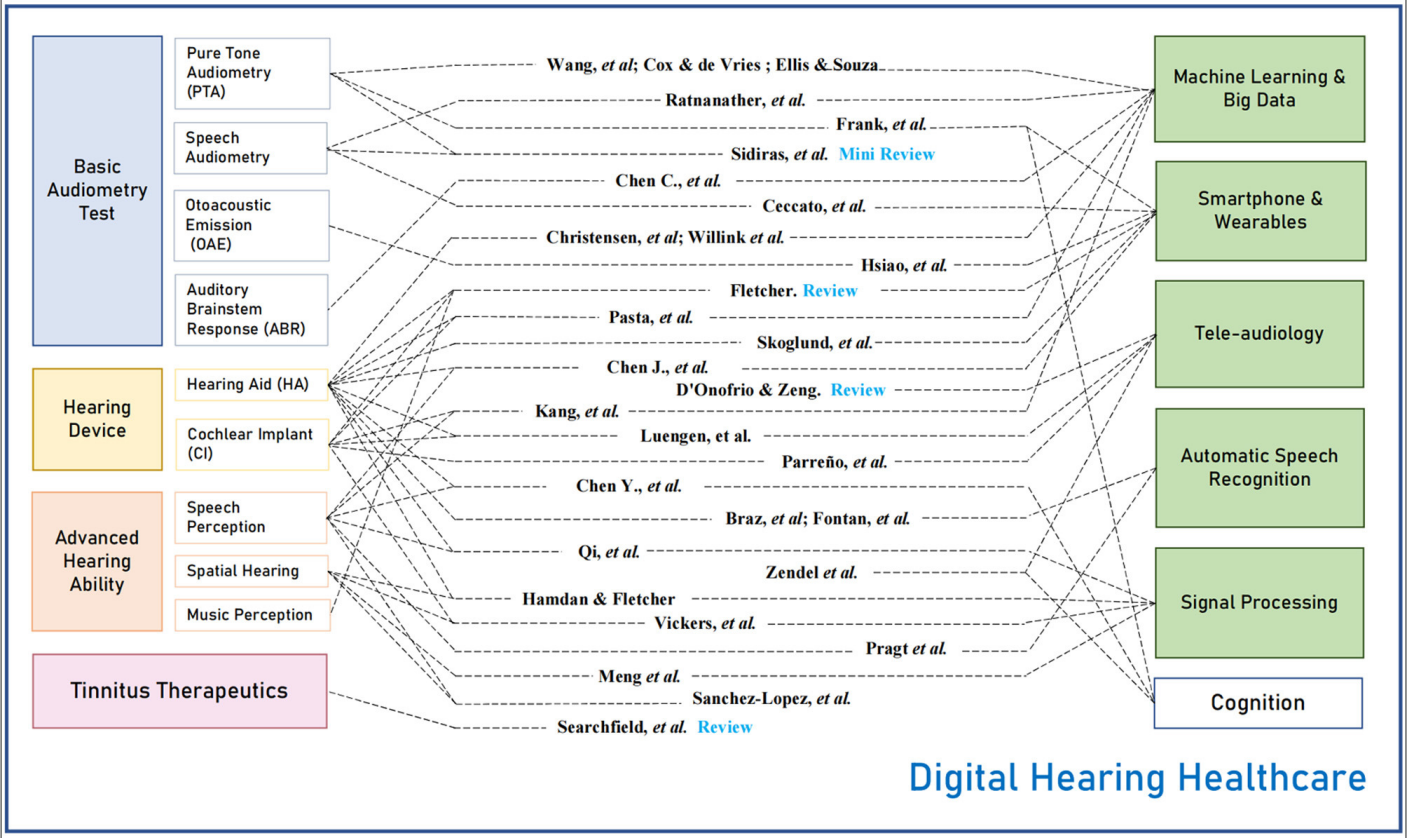
Distribution of the 30 articles in this Digital Hearing Healthcare (DHH) Research Topic.

## Basic audiometry testing

Audiometry consists of methods used in a standard audiology center to identify and quantitatively measure the degree and pathogenesis of hearing loss. Pure-tone audiometry (PTA) is the most widely used method, resulting in a quantitative estimate of the audiogram. The audiogrm reflects the hearing threshold as a function of frequency (2). Wang et al. studied phenotypes of noise-induced hearing loss by clustering analysis on audiograms of more than 10 thousand shipyard employees. Cox and de Vries proposed an improved probabilistic model-based PTA procedure that combines more prior information about the patients. This line of research on machine learning-based methods is expected to facilitate automatic PTA by shortening the testing time while maintaining accuracy (3, 4). Ellis and Souza examined the performance of a previously patented method for measuring an audiogram without precise sound level calibration in remote testing. That method combined the audiometric slope between pure-tone thresholds estimated at 2 and 4 kHz and questionnaire information. The latter two methods were examined in simulation experiments based on large databases. Frank et al. validated an iPad-based PTA app in 25 individuals with mild cognitive impairment and mild dementia. Beyond PTA, speech audiometry is critical for assessing the ability to hear and comprehend speech. Ratnanather et al. developed an automated program to calculate the phoneme confusion pattern based on the records from word or sentence-level tests. Ceccato et al. developed a French version of the antiphasic digits-in-noise (DIN) test for smartphone hearing screening. DIN, which does not require precise sound level calibration and tests digit-triplet reception thresholds in noise, has been developed in many languages. The antiphasic DIN may be more sensitive to unilateral hearing loss and conductive hearing loss than traditional diotic DIN (5). Other than the behavioral tests, novel technologies for otoacoustic emission (OAE) and auditory brainstem response (ABR) detection were also studied. Hsiao et al. introduced the stimulus design and signal processing to measure distortion-product OAE with a single loudspeaker in the ear. The research targets intelligent consumer earphones with integrated hearing health monitoring functions. Chen C. et al. proposed a machine learning method to recognize ABR waveforms automatically, and the results showed its feasibility in saving time and helping make diagnoses.

### Hearing aid

As one of the most widely-used treatments for hearing impaired (HI) listeners, hearing aids (HAs) were explored and discussed adequately in this specific issue, including more than ten papers in which HA-related research was conducted. Willink et al. focused on the alternative pathway for hearing care by examining the HI population who do not use HAs via describing their characteristics and health care utilization patterns with the sample size of 7,361 Medicare beneficiaries. Two papers (Pasta et al.; Christensen et al.) tried to provide a deeper insight into the adoption of hearing care treatments and individual HA usage patterns by analyzing objective HA use data logged from real-world users. Braz et al. and Fontan et al. developed new fitting methods combining automatic speech recognition (ASR) to optimize compression parameters for HA. One of the most important objectives of using HAs is to improve the speech perception of HA customers, and the perception of the speech signals processed by HA technology is probably related to listeners' language experience (6). Three papers investigated the speech perception of HA users speaking Mandarin Chinese. Qi et al. aimed to examine the effects of an adaptive non-linear frequency compression algorithm on speech perception and sound quality in native Mandarin-speaking adult listeners with sensorineural hearing loss. They found significant perceptual benefit from the adaptive non-linear frequency compression algorithm in detecting high frequency sounds at 8 kHz, in consonant recognition, and in an overall sound quality rating. Chen J. et al. aimed to evaluate the improvement of speech recognition in noise measured with signal-to-noise ratio by using wireless remote microphone technology for HI listeners speaking Mandarin Chinese. The experiment results confirmed the significant improvement. Chen Y. et al. investigated the relationships between cognitive and hearing functions in older Chinese adults with HAs and untreated hearing loss. They concluded that speech perception in noise is significantly associated with different cognitive functions. In addition to speech perception, researchers also tried to extend HA functions to make HI listeners' hearing experience close to normal hearing, such as spatial hearing and music perception. Hamdan and Fletcher proposed a compact two-loudspeaker virtual sound reproduction system for clinical testing of spatial hearing with hearing-assistive devices. They found such a system can give broad access to advanced and ecologically valid audiological testing, and it could substantially improve the clinical assessment of hearing-assistive device users. Meng et al. investigated a novel minimum audible angle (MAA) test using virtual sound source synthesis and found it to be a suitable alternative to more complicated and expensive setups. Sanchez-Lopez et al. developed the Better hEAring Rehabilitation (BEAR) project to measure various hearing abilities, especially supra-threshold hearing deficits.

### Cochlear implant

For most patients with severe-to-profound hearing impairment, HAs cannot provide enough benefits for their speech communication. The cochlear implant (CI) is a good option for them to partially (re)gain speech perception abilities, at least in a quiet environment. However, CI users still face significant challenges in advanced hearing functions, e.g., speech-in-noise perception, spatial hearing, and music perception (7). Many efforts have been made to improve the CI performance on these issues gradually. Three papers in this Research Topic researched CI improvements. Kang et al. used recent progress to improve noise suppression or speech enhancement of CIs and found that the intelligibility of the denoised speech can be significantly enhanced when a neural network is trained with a loss function bias toward more noise suppression than that with equal attention on noise residue and speech distortion. Vickers et al. developed a package of virtual-reality games to train spatial hearing in children and teenagers with bilateral CIs. The virtual-reality implementation demonstrated to be more engaging than traditional rehabilitation methods. As introduced in the HA section, Fletcher reviewed the effects of haptic stimulation methods on enhancing music perception in HA and CI listeners. New technologies such as advanced algorithms, virtual reality, and multimodal stimulation should be up-and-coming to positively integrate future CI devices with CI users' lives.

### Tinnitus

Tinnitus, or ringing in the ears, affects 15% of the general population. At present, tinnitus mechanisms remain unclear (8). Although no cure is available for tinnitus, safe and effective therapy and counseling have been developed to help alleviate its symptoms. Searchfield et al. reviewed the first three generations of digital technologies for tinnitus management, ranging from digital hearing aids and apps to stand-alone, customized digital devices. Most interestingly, they forecast the fourth-generation digital technology that incorporates physiological sensors, multi-modal transducers, and AI for personalized tinnitus therapy.

### Tele-audiology

Accelerated by COVID-19, tele-audiology has become a necessary means of delivering hearing care service, especially for the most-vulnerable elderly population. D'Onofrio and Zeng examined technological and regulatory barriers for a wide range of audiological services from audiometry to hearing device fitting and rehabilitation. Most of these barriers can be overcome not only to provide reliable and effective tele-audiological service, but more importantly, to improve access to care, increase follow-up rates, and reduce travel time and costs. Luengen et al. envisioned an innovative tele-audiological model that consists of (1) one-to-one remote interaction between a patient and an audiologist, (2) a one-to-many model that relies on automated service provided by AI, and (3) a several-to-many application that can fit hearing devices not only based on the patient's audiological profile but also their listening environments. One critical piece of information in tele-audiology is reliable monitoring of hearing status and device functionality, especially for a complicated device such as a CI. Parreño et al. developed a self-monitoring method of measuring CI electrode impedance. Making use of a computer, the device interface provided by the manufacturer, and secure internet connectivity, they were able to record, transfer and monitor impedance at home without any adverse events. Another critical piece of information is accurate and reliable communication between the patient and providers in tele-audiology. Zendel et al. found that current teleconferencing is less reliable than in-person instruction in terms of patients' recollection of the healthcare messages delivered. Speech and video quality, as well as communication methods, need to be improved in order to reduce the memory deficit associated with current telehealth technologies.

## Machine learning and big data

Machine Learning (ML) represents a set of tools that reveal complex trends in data that would be difficult or impossible to discern otherwise. Often these tools are powered by large amounts of data (“big data”), which provide more opportunities to observe interesting trends. Ellis and Souza took this approach to train an ML classifier of audiograms using almost 10,000 individuals from a large national auditory and demographic information database. Wang et al. followed a similar approach, analyzing over 10,000 audiograms for notch appearance, identifying three noise-induced hearing loss subtypes in the process. Using a different type of data, Chen C. et al. employed a recurrent neural network and signal processing to recognize potential waveforms in Auditory Brainstem Response (ABR) signals. Big Data can be leveraged in different ways, as well. For example, Cox and de Vries learned from existing databases how to shorten the test time of future audiograms computed in real time using an ML algorithm.

Ongoing data collection enabled by always-connected devices offers the opportunity to trade up for more informative algorithms as more data become available. Christensen et al. clustered the usage activity of 64 hearing aid users over several days with a straightforward K-means algorithm. Pasta et al. approached a similar problem with nearly 16,000 users and several month's worth of data. They used a more sophisticated multilayer neural network to reveal finer trends.

ML methods have great potential for therapeutics as well as diagnostics. Kang et al. describe a deep learning (i.e., many-layered neural network) approach to improve speech enhancement in cochlear implant (CI) encoding algorithms. Braz et al. used knowledge of audiograms and a genetic algorithm to search the many configurations of hearing aid program settings to optimize the device for a particular patient.

### Smartphone and wearables

Miniaturization has led to smaller sensors and stimulators to create haptic devices worn as gloves or bracelets. Fletcher contributed a review paper to discuss “Can haptic stimulation enhance music perception in HI listeners.” It has been reported that “electro-haptic stimulation” improves melody recognition and pitch discrimination, as well as speech-in-noise performance and sound localization. This review paper focused on the use of haptic stimulation to enhance music perception in HI listeners. One of his conclusions is that haptic devices, in concert with other modalities, can enhance the music experience in hearing impaired listeners. Using several sensor technologies, modern HAs strive to become better, more personalized, and self-adaptive devices that can handle environmental changes and cope with the day-to-day fitness of the users. Skoglund et al. measured the accuracy of activity tracking, e.g., step detection, through small accelerometers embedded in hearing aids. They showed classification of activities was similar to conventional activity tracking techniques, which is encouraging for applications in hearing health care. In noisy conditions, small microphones can be employed to improve the signal-to-noise ratio. Chen J. et al. confirmed that wireless microphones improve speech recognition in Chinese hearing impaired listeners when the target speaker is at a larger distance.

### Automatic speech recognition

Within digital hearing health care, ASR has various applications, including the use of ASR as a tool to understand conversations. Pragt et al. examined the speech recognition performance of four ASR apps on smartphone using conventional Dutch audiological speech tests. They compared human speech recognition performance and performance by ASR apps. They found that the performance of the apps was limited on audiological tests that provide little linguistic context or use low signal-to-noise levels. They concluded that conventional performance metrics and conventional hearing tests are insufficient to assess the benefits of ASR apps for the deaf and proposed that adding new performance metrics including the semantic difference as a function of SNR and reverberation time could help monitor ASR performance. Another strategy uses ASR, hearing loss models, and hearing aid signal processing simulations to mimic impaired hearing listeners. As mentioned in the Hearing Aid section, Braz et al. and Fontan et al. demonstrated that ASR, in combination with random search algorithms, can be used to find optimal subsets of parameter settings for hearing aids. These optimizations should subsequently be validated in actual hearing aid users, especially in case of severe hearing loss. Recently, ASR has also been used in predicting CI performance (9) and, together with the above HA studies, demonstrates good potential in modeling hearing device performance.

## Discussion and future outlook

It is worth noting that several articles present the progress of their projects driven by long-term interdisciplinary collaborations. They are SHOEBOX Audiometry for hearing screening using sound-attenuating headphones (Frank et al.), Better hEAring Rehabilitation (BEAR) to provide a test battery for hearing loss characterization (Sanchez-Lopez et al.), User-Operated Audiometry (UAud) to introduce an automated system for user-operated audiometric testing into everyday clinical practice (Sidiras et al.), BEARS (Both EARS) to develop virtual reality training suite for improving spatial hearing for 8–16 year-olds with bilateral CIs (Vickers et al.), OPRA for developing Objective Prescription Rule based on ASR (Braz et al.; Fontan et al.). These long-term, collaborative projects may result in improved efficiency while lowering the cost for hearing healthcare.

The interdisciplinary nature of the DHH field also provides great challenges and opportunities for both academic research and industrial development. For example, the Interdisciplinary Technologies for Auditory Prostheses (iTAP) conference series (http://www.itap.org.cn/) was founded in 2017 in China and has a vast interest overlap with this DHH Research Topic. Computational audiology, the application of complex models to clinical care (10), is an important part of digital hearing health care. Many contributors to this Research Topic first discussed their projects at the Virtual Conferences on Computational Audiology (VCCA) (https://computationalaudiology.com/), which have provided a platform to share progress in e-research, machine learning, big data, models, virtual reality, and other developments. The Research Topic, iTAP, and VCCA all share the same goal of providing a platform for facilitating communication between experts from different fields and accelerating research and development.

In conclusion, the contributions in this Research Topic have demonstrated that novel digital technologies in machine learning, big data, signal processing, telehealth, and mobile health are being actively applied toward hearing healthcare applications. Improving the accessibility and performance of audiometry, hearing-assist devices and tinnitus therapeutics stands out as successful application of these technologies. This Research Topic provides general readers a glimpse of the emerging Digital Hearing Healthcare field and hopefully will inspire more people, companies, and organizations to develop and deploy digital health techniques for better hearing, and as a result, a better world.

## Author contributions

All authors except J-WW were guest associate editors of the Research Topic. J-WW has made significant contributions to the call-for-paper through the VCCA network. All authors wrote the paper text and approved the submitted version.

## Funding

QM was supported by Guangdong Basic and Applied Basic Research Foundation Grant (2020A1515010386), Science and Technology Program of Guangzhou (202102020944). JC was supported by the National Key Research and Development Program of China (2021ZD0201503) and National Natural Science Foundation of China (12074012). CZ was supported by National Natural Science Foundation of China (31900801). DB was supported by the Advanced Education Research & Development Fund and the National Institues of Health (R21EY033553). F-GZ was supported by the National Institues of Health (5R01DC015587 and 1R01AG067073).

## Conflict of interest

DB reports equity ownership in Bonauria. F-GZ reports equity ownership in Axonics, DiaNavi, Neocortix, Nurotron, Syntiant, Velox and Xense. The remaining authors declare that the research was conducted in the absence of any commercial or financial relationships that could be construed as a potential conflict of interest.

## Publisher's note

All claims expressed in this article are solely those of the authors and do not necessarily represent those of their affiliated organizations, or those of the publisher, the editors and the reviewers. Any product that may be evaluated in this article, or claim that may be made by its manufacturer, is not guaranteed or endorsed by the publisher.
